# The Forty-Eighth Annual Commencement of the Baltimore College of Dental Surgery

**Published:** 1888-04

**Authors:** 


### Editorial, Etc.

THE FORTY-EIGHTH ANNUAL COMMENCEMENT OF THE BALTIMORE COLLEGE OF DENTAL SURGERY was held at the Lyceum Theater, Baltimore, Md., on Thursday, March 8th, 1888.

The annual oration was delivered by Dr. W. H. Dwinelle, and the valedictory by W. W. Dunbracco, D. D. S.

The number of matriculates was one hundred and fourteen.

The degree of D. D. S., was conferred upon the following graduates by Professor R. B. Winder, dean of the faculty:

| | |
|---|---|
| D. S. Arnold, | Alabama |
| R. Blackwell, | Virginia |
| R. H. Blair, | Texas |
| F. V. Brooking, | Illinois |
| C. C. Buck, | Maryland |
| W. E. Bunn, | Georgia |
| W. D. Cowan | Canada. |
| J. H. Crossland, | Alabama |
| J. C. Dana, | New York |
| M. L. Dawson, | Virginia |

| | |
|---|---|
| W. W. Dunbracco, - - - | Maryland |
| J. W. Fisher, - - - - | Virginia |
| J. D. Ford, Jr., - - - | Maryland |
| W. S. Gregory, - - - | Virginia |
| S. W. Gregory, - - - | N. Carolina |
| C. F. Harding, - - - | New York |
| G. E. Hardy, - - - | Virginia |
| C. W. F. Holbrook - - - | New Jersey |
| W. F. Holt - - - - | Georgia |
| T. H. Kellam, - - - - | Virginia |
| A. E. Kellogg, - - - | Pennsylvania |
| E. C. Kirby, - - - - | Maryland |
| W. R. Knight, Jr., - - - | New York |
| L. P. Leonard, - - - | Dakota |
| A. C. Liverman, - - - | N. Carolina |
| B. F. Mardis, - - - | Pennsylvania |
| C. H. McLean, - - - | Illinois |
| A. Mills, - - - - | Canada |
| R. H. Moloney, - - - | Canada |
| W. P. Moore, - - - - | Virginia |
| C. G. Myers, - - - | Indiana |
| H. Muller, - - - - | Germany |
| J. M. Parker, - - - | N. Carolina |
| G. W. Patten, - - - - | Minnesota |
| W. H. Phillips, - - - | New York |
| J. Rust, - - - - | Virginia |
| W. H. Savage, - - - | N. Carolina |
| J. W. Semones, - - - | Virginia |
| A. W. Seidler, - - - | Maryland |
| J. W. Smith, - - - - | Virginia |
| M. A. Sparks, - - - | Alabama |
| G. J. Sproul, - - - - | Canada |
| R. H. Stephenson, - - - | Virginia |
| S. Szuwalski, - - - - | Maryland |
| H. W. Talley, - - - | Virginia |
| W. J. Thurmond, - - - | Georgia |
| J. B. Walton, - - - | D. Columbia |
| J. E. Ward, - - - | Pennsylvania |
| F. A. Warnes, - - - | Connecticut |

| | |
|---|---|
| W. D. Williams. - - - | Virginia |
| L. D. Wilson, - - - | Virginia |
| J. T. Wright, Jr., - - - | Virginia |
| R. E. Wilkinson, - - - | New York |

